# Developing the WHO Mosaic Toolkit to End Stigma and Discrimination in Mental Health: A participatory approach

**DOI:** 10.1007/s40211-025-00536-4

**Published:** 2025-08-25

**Authors:** Petra C. Gronholm, Jay-Bethenny Gallimore, Ledia Lazeri, Jason Maurer, Maria Milenova, Arnaud Poitevin, Cassie Redlich, Ana Maria Tijerino Inestroza, Zbyněk Roboch, Graham Thornicroft

**Affiliations:** 1 Centre for Global Mental Health, Institute of Psychiatry, Psychology, and Neuroscience, King’s College London, London, UK; 2 Centre for Global Mental Health, Department of Population Health, London School of Hygiene and Tropical Medicine, London, UK; 3 World Health Organization Regional Office for Europe, Copenhagen, Denmark; 4 Centre for Global Mental Health and King’s Improvement Science Centre, Institute of Psychiatry, Psychology, and Neuroscience, King’s College London, London, UK; 5 ESPER Pro, Marseille, France; 6 Department of Public Mental Health, National Institute of Mental Health, Klecany, Czech Republic; 7 Global Mental Health Peer Network, https://www.gmhpn.org/

**Keywords:** Stigma reduction interventions, Social contact, Coproduction, Lived experience, Global mental health, Stigma vermindernde Interventionen, Sozialkontakt, Koproduktion, Erlebte Erfahrung, Psychische Gesundheit im globalen Kontext

## Abstract

Stigma and discrimination related to mental health are major global challenges that demand urgent, evidence-based responses. The Mosaic Toolkit to End Stigma and Discrimination was developed in response to calls for practical guidance to end stigma and discrimination. This article outlines the co-production process behind the toolkit’s creation, carried out from December 2022 to August 2024, through a collaboration between World Health Organization (WHO) Regional Office for Europe, the Global Mental Health Peer Network, and King’s College London. The development involved extensive consultation with individuals with lived experience and a wide range of stakeholders, ensuring cultural relevance, inclusivity, and applicability in diverse settings. The toolkit outlines core principles for stigma reduction, an action-oriented process model, illustrative case studies, and supportive resources including advocacy tools. Grounded in the principle of “nothing about us without us” and the power of social contact, the practical strategies within the WHO Mosaic Toolkit offers a concrete path to ending mental health stigma. The next crucial step will involve supporting implementation in real-world contexts to create lasting change. Designed for global application, the toolkit aims to foster dignity, inclusion, and rights-based approaches to end mental health stigma and discrimination.

## Abstract (de)

Stigmatisierung und Diskriminierung im Zusammenhang mit psychischer Gesundheit sind große Herausforderungen weltweit, sie erfordern dringende, evidenzbasierte Antworten. Das Mosaik-Toolkit zur Beendigung von Stigmatisierung und Diskriminierung wurde als Reaktion auf die Forderung nach einer praktischen Anleitung zur Beendigung von Stigmatisierung und Diskriminierung entwickelt. Im Beitrag wird der Koproduktionsprozess zur Erstellung des Toolkits beschrieben, der von Dezember 2022 bis August 2024 in einer Zusammenarbeit zwischen dem Regionalbüro der Weltgesundheitsorganisation (WHO) für Europa, dem Global Mental Health Peer Network und dem Kingʼs College London realisiert worden ist. Seine Entwicklung beinhaltete umfassende Konsultationen von Menschen mit „lived experience“ (erlebte Erfahrung) und einer Vielzahl weiterer Stakeholder, um kulturelle Relevanz, Inklusivität und Anwendbarkeit in vielfältigen Kontexten sicherzustellen. Das Toolkit entwirft essenzielle Prinzipien für den Abbau von Stigmatisierung, es enthält ein handlungsorientiertes Prozessmodell, klar verständliche Praxisbeispiele und unterstützende Ressourcen, u. a. auch Instrumente für die Interessenvertretung. Basierend auf dem Prinzip „Nichts über uns ohne uns“ und auf der Kraft von Sozialkontakten bieten die praktischen Strategien des WHO-Mosaik-Toolkits einen konkreten Weg zur Überwindung von Stigmatisierung im Bereich der psychischen Gesundheit. Der nächste wesentliche Schritt wird darin bestehen, die Implementierung in Real-World-Kontexten zu unterstützen, um nachhaltige Veränderungen zu erreichen. Das Toolkit ist für die globale Anwendung konzipiert und soll Würde, Inklusion und auf Rechten basierende Ansätze fördern, um Stigmatisierung und Diskriminierung im Bereich psychische Gesundheit zu beenden.

## Background

Mental health-related stigma and discrimination are pressing global challenges [1]. Stigma plays a major role in reinforcing social exclusion, and people with mental health conditions often face discrimination across various areas of life, leading to isolation and disconnection from their social networks [2, 3]. This exclusion can worsen mental health, creating a cycle of silence and shame. Stigma also discourages individuals from seeking help, out of fear of judgment or being labelled, delaying access to support and treatment and potentially worsening their condition. Tackling stigma is essential for promoting inclusion and timely mental health care.

Effective stigma-reduction strategies are grounded in social contact [4], and the principle of “nothing about us without us”, highlighting the importance of involving people with lived experience [5]. Social contact—whether direct or indirect—can reduce prejudice by fostering empathy, perspective-taking, and lowering anxiety, while challenging negative stereotypes [6, 7]. Furthermore, involving people with lived experience ensures their insights shape efforts and that their needs and priorities are centred in the process [1].

Recognising both the urgency of addressing stigma and the strong evidence for how to do so, the Lancet Commission on Ending Stigma and Discrimination in Mental Health [1] has called for a practical, evidence-based toolkit to guide stigma-reduction efforts. The Mosaic Toolkit to End Stigma and Discrimination [8] was developed in response to this call, as a ‘global good’ [9] intended to provide an accessible tool for use at scale to tackle the global health challenge of mental health stigma and discrimination. It supports anyone interested in anti-stigma work, especially those with little prior experience. It also serves as a helpful resource for experienced advocates seeking stakeholder support or expanding stigma-reduction efforts into new areas.

This article describes the development process of the World Health Organization (WHO) Mosaic Toolkit, and outlines the key evidence-based principles and implementation strategies provided within it.

## Approach and procedure

### Participatory co-production approach and writing process

A participatory co-production approach was used to develop the toolkit, involving close collaboration with diverse collaborators and stakeholders to ensure it is relevant, inclusive, and grounded in real-world needs and experiences.

The core writing group included representatives from the WHO Regional Office for Europe (WHO-EURO), the Global Mental Health Peer Network, and King’s College London. This collaboration brought together lived experience expertise, leadership in global health, and academic knowledge on stigma and discrimination. Oversight and additional input were provided by a working group comprising WHO pan-European Mental Health Coalition members, international stigma experts, and global lived experience representatives.

Developed between December 2022 and August 2024, the toolkit was shaped through in-person and online workshops where the writing group collaboratively defined its scope and structure. Between workshops, writing responsibilities were shared, using online documents to support open collaboration and transparency. Content was reviewed, discussed, and edited in regular online meetings. At key points, the broader stakeholder group was consulted for critical feedback and technical input on the toolkit’s content and direction. These consultations are detailed next.

### Stakeholder consultations

1. Working group online workshop

Two half-day online workshops were held on 30–31 August 2023 with the wider working group to review an initial draft of the toolkit’s structure and content. Forty international participants (including stigma researchers, people with lived experience, academics, WHO staff, and policy makers) were invited to ensure diverse professional and lived perspectives, as well as broad geographic representation.

Each workshop session involved small group discussions facilitated by writing group members. Participants reviewed two phases of the proposed stigma reduction process per day, providing input on content relevance, contextual challenges, and practical improvements. Suggestions for illustrative case studies, frequently asked questions (FAQs), and resources for inclusion were collected using collaborative online tools supplemented by facilitators’ notes.

Workshop feedback was reviewed by the writing group, and key themes were integrated into the subsequent toolkit draft. This included emphasising the central role of lived experience, the importance of adapting activities to local context, and working with community stakeholders for sustainability. Nearly one hundred questions were proposed for the FAQ section and screened for inclusion. Participants also contributed ideas for distributing the toolkit.

2. WHO pan-European Mental Health Coalition Workshop

A second workshop was held in Brussels, Belgium on 22–23 May 2024, as a part of a larger meeting of the pan-European Mental Health Coalition and the European Union Joint Action on Implementation of Best Practices in the area of Mental Health (JA ImpleMENTAL).

This workshop session was co-led by writing group members from the Global Mental Health Peer Network and WHO-EURO, to reinforce the important principle of co-creation with people with lived experience. A draft version of the toolkit was shared with participants, and its basic structure was presented. Input was sought to further refine the toolkit through feedback on the content flow, and what was perceived as essential elements to help the writing group to condense the text. Participants also provided suggestions of how they might use the toolkit in their context, to support the development of the toolkit distribution strategy.

3. Global Mental Health Action Network (GMHAN) online consultation

A final consultation of the toolkit was conducted with the GMHAN Stigma and Discrimination Working Group on 26 June, 2024, attended by 33 members from across Europe, Africa, Asia, Oceania, and South America.

The near-final toolkit was presented, focusing on its scope, key principles, and intended use. Attendees were consulted on how they envisioned the toolkit could be used in their context, and whether they had further resources to suggest for inclusion in appendices. Questions from the meeting were also considered for the toolkit’s FAQ section (e.g. on the toolkit’s adaptability for digital delivery, integration into national programs, and relevance for specific issues such as substance use disorders, suicide prevention, and intersectional stigma).

### Case study development

As the toolkit was designed to be a resource supporting various anti-stigma activities and programmes, it was decided it would include a range of case studies to demystify anti-stigma work and demonstrate its practical relevance across various projects with different scope, specific aims, and across implementation contexts.

Eleven case study domains were included, based on the writing group members’ experience, and feedback from the stakeholder consultations: workplace, healthcare, schools/youth, low-resource settings, cultural adaptation, grassroots initiatives, individual-level action, national campaigns, WHO-EURO regional priorities (Eastern Europe and Central Asia), structural change, and media.

Interviews with case study representatives were conducted between February and April 2024. The interviews focused on understanding how the implementation processes emphasised in the toolkit had been achieved in the case study projects. Interview transcripts were synthesised into summaries highlighting key elements of each anti-stigma initiative. These were reviewed and approved by the case study representatives before inclusion in the final toolkit.

Besides case studies, the toolkit also includes a spotlight on policies and actions addressing stigma and discrimination in mental health across the European Union, showcasing international efforts in stigma reduction.

## Toolkit design and contents

The final toolkit is structured into four main sections. It begins with the rationale and principles for reducing mental health stigma and discrimination, followed by a process model outlining how to take action, and the case studies demonstrating how these processes work in practice. The final section revisits key principles and offers concluding remarks.

Appendices include: 1) FAQs; 2) links to further resources on stigma, social contact, lived experience, rights, recovery, evaluation, WHO tools, and peer communities; 3) discussion of rights-based and disability perspectives; and 4) advocacy talking points on common myths and misconceptions.

### Overview of core principles of the WHO Mosaic Toolkit

The toolkit highlights three key principles for reducing stigma and discrimination: leadership or co-leadership by people with lived experience, social contact, and inclusive partnerships.

People with lived experience should be involved from the start, ideally in leadership roles where their contributions are valued equally to other expertise. This participation must be meaningful, rather than tokenistic, and should prioritise safety and empowerment. It includes being treated with dignity, recognising lived experience as equal to professional knowledge, and addressing power imbalances. Safe spaces are essential, and diverse experiences and identities should be embraced as strengths, not generalised.

Social contact is supported by strong evidence for reducing stigma through fostering empathy, perspective-taking, and reducing anxiety. The toolkit outlines key features for effective social contact (direct or indirect) [4], such as personal stories, challenging stereotypes, highlighting recovery, ensuring equal status, and working toward shared goals. Appropriate elements of social contact should be included in all anti-stigma activities.

Inclusive partnerships mean working not only with people with lived experience, but also with a broad range of stakeholders—such as intervention target groups, community leaders, healthcare providers, non-governmental organisations (NGOs), policymakers, media, researchers. These collaborations ensure activities are locally relevant and impactful. Media can broaden reach, researchers support evaluation, and government involvement aids sustainability. Peer-led groups help empower others and spread learning.

### Implementation process model

The WHO Mosaic Toolkit’s four-step process model for reducing stigma and discrimination is a flexible guide rather than a strict checklist. It should be adapted to each activity’s goals, resources, and cultural context. Stigma varies across settings, so efforts must reflect local realities, drivers of change, and available tools. The core principles—lived experience leadership, social contact, and inclusive partnerships—should inform all stages of the process.

*Step 1: Identify and define aims. *Effective anti-stigma work begins with a clear aim and an understanding of context. This includes defining the type of stigma being addressed, identifying the target group, and outlining what success looks like. The section covers how to frame aims, engage lived experience, and build partnerships.

*Step 2: Plan and prepare. *This stage focuses on designing and adapting activities, planning monitoring and evaluation, and building implementation readiness.

*Step 3: Launch and learn. *With activities underway, the emphasis shifts to implementation, coordination, and adaptation. Topics include communication, peer support, use of online platforms, and ensuring safety for all involved. This step highlights the need for ongoing reflection and adjustment.

*Step 4: Reflect and proceed. *As activities conclude, it is important to reflect on outcomes and decide whether to continue, expand, or close the project. This step also underscores responsible wrap-up, sharing lessons learned, and building momentum for future efforts.

### Exemplifying case studies

Table 1 provides an overview of the case studies included in the toolkit, exemplifying how the principles and processes can be used to effectively address stigma and discrimination in diverse contexts and communities.

**Table 1:** Overview of case studies included in the Mosaic Toolkit

Programme/activity	Setting	Case study domain	Summary
Ben Ogden	UK	Individual-level action	Ben Ogden stood in cities and towns across the UK, sharing his mental health experience to encourage people to speak out about their mental health and destigmatise mental health conversations
Facettes Festival	France	Schools/youth	A youth-focused mental health festival aimed to raise awareness and reduce stigma through creative, community-centred engagement
Bearapy	China	Workplace	A programme aimed to reduce stigma in the workplace, emphasizing social contact and language framing, positioning mental health as a critical component of employee resilience and well-being
Living Libraries	France	Grassroots initiative	An initiative bringing people with lived experience to converse and share their stories with members of the public, humanizing mental health experiences, fostering empathy, and dismantling stereotypes
Yellow September	Iceland	National campaign	A national anti-stigma and suicide prevention campaign encouraging public dialogue about mental health and normalizing help-seeking behaviour through widespread visibility
Speak Up	Kenya	Media	An initiative aimed at reshaping public attitudes and transforming how mental health is portrayed in the media through public awareness campaigns and journalist training
Responding to Experienced and Anticipated Discrimination Mental Health (READ-MH)	Tunisia	Healthcare	A training initiative for mental health professionals designed to raise awareness of, and reduce stigma, within healthcare settings
Systematic Medical Appraisal, Referral and Treatment Mental Health (SMART-MH)	India	Cultural adaptation	A community-based programme in rural India, aimed at improving the screening, identification, and management of common mental health conditions
Na Rovinu [On the level]	Czechia	WHO-EURO regional priority (Eastern Europe and Central Asia)	An initiative promoting mental health literacy and stigma reduction through media storytelling, peer support groups, and sustainable system change
EN AF OS [One of us]	Denmark	National campaign	A national campaign whereby “Ambassadors” — individuals with lived experience — are trained to share their stories with diverse target groups to raise awareness and promote inclusion across the country
Taskeen	Pakistan	Structural change	A policy advocacy campaign that successfully led to the decriminalization of suicide in Pakistan

### Launch event

The WHO Mosaic Toolkit was officially launched on World Mental Health Day 10 October 2024, at the UN City in Copenhagen, Denmark. The launch was attended by the toolkit’s contributors, along with key collaborators and stakeholders, including the WHO Europe Regional Director, WHO Europe Director of the Division of Country Health Policies and Systems, WHO Europe Regional Advisor for Mental Health, and WHO Headquarter’s Director of Mental Health, Substance Use and Brain Health. Lived experience was also centred at the launch event, through a personal narrative by one of the Mosaic Toolkit writing group members representing the Global Mental Health Peer Network.

The launch featured a presentation of the toolkit’s purpose and development, core principles, structure and content, and its featured case studies. This was followed by a panel discussion with global experts in the field of mental health stigma and discrimination, and representatives from key advocacy organisations and other stakeholders. The launch event reaffirmed the WHO Mosaic Toolkit’s call to action: to advocate for and implement effective strategies to end stigma and discrimination in mental health.

## Discussion

The WHO Mosaic Toolkit provides practical guidance on how to end stigma and discrimination in mental health. It represents a critical, evidence-based contribution to mental health systems worldwide. Grounded in participatory co-production, it is designed as a scalable, open-access resource supporting broad uptake across diverse settings. The toolkit is intended for a wide audience from individuals to organisations, aiming to simplify stigma reduction for those new to anti-stigma work as well as experienced advocates.

The toolkit’s focus on lived experience leadership, social contact, and inclusive collaboration responds directly to longstanding challenges in embedding rights-based, equitable approaches. For example, it aligns with the UK’s proposed Mental Health bill [HL] 2024–25 [10] which seeks to centre rights, dignity, and autonomy in care, and WHO’s introduction of stigma indicators in its 2024 Mental Health Atlas [11]. Also, in 2024 the EU Commission set up a drafting group to support EU Member States in tackling mental health stigma and discrimination [12], which has developed an EU support package including actions such as an EU spotlight section in the Mosaic Toolkit.

The toolkit’s development process actively demonstrated co-leadership by people with lived experience. The core writing group included representatives from the Global Mental Health Peer Network, who were fully involved in shaping the toolkit’s structure, writing and refining content, identifying and interviewing case studies, and engaging other professionals with lived experience, such as content reviewers and illustrators. Importantly, all contributions were fairly compensated, reflecting both ethical practice and recommendations for nontokenistic involvement [13, 14].

Lived experience members also played a key role in stakeholder consultations, adding a dimension of social contact that showcased recovery in action and demonstrated that people with mental health conditions are capable and valued collaborators. Such involvement can also be empowering for individuals who may have once internalised stigma, and it strengthens advocacy organisations through leading by example.

Developing the toolkit’s scope and content brought challenges and valuable discussions. A key task was balancing broad applicability with relevance to specific projects, target groups, stigma types, and local cultural contexts. The final process model aims to be flexible enough for global adaptation while remaining locally useful. The selected case studies highlight how the toolkit’s principles can be applied across diverse settings and initiatives.

A key next step will be helping stakeholders apply the toolkit in real-world systems. Since its launch, the WHO Mosaic Toolkit has become one of WHO-EURO’s most downloaded resources, reflecting strong global interest. Two main challenges have emerged: understanding how users engage with WHO resources, and creating a pathway to professionalise lived experience leadership. Further consultations with policymakers, civil society, and people with lived experience could help identify barriers, assess system needs, and co-design uptake strategies—insights that will shape WHO’s ongoing policy support. The toolkit is currently being translated into French and Italian, but further translations would facilitate its uptake.

To ensure lasting impact, future research should examine how the toolkit’s impact, and how it is adapted across contexts, how its principles are implemented in practice, and what supports its sustained use. Assessing the toolkit’s effect on structural stigma, especially in healthcare and policy, will be vital to ensure it drives real, systemic change.

## Conclusion

In recent years there has been a remarkable increase in research on interventions to reduce stigma and discrimination in the field of mental health. This means that the evidence is now clear that social contact is the foundation of effective anti-stigma interventions. Under the leadership of the mental health team at WHO-EURO, a new practical approach has been developed to put this evidence into practice: the WHO Mosaic Toolkit. It has been carefully designed to be relevant, with appropriate adaptation for different contexts and cultures, to be used worldwide to make an important contribution to ending stigma and discrimination for good.
